# Establishing Minimum Criteria for Stem Cells from Human Exfoliated Deciduous Teeth (SHEDs) Cultured in Human Platelet Lysate (hPL)-Contained Media as Cell Therapy Candidates: Characterization and Predictive Analysis of Secretome Effects

**DOI:** 10.3390/cells14040316

**Published:** 2025-02-19

**Authors:** Ji-Young Yoon, Bình Do Quang, Ji-Sun Shin, Jong-Bin Kim, Jun Hee Lee, Hae-Won Kim, Jung-Hwan Lee

**Affiliations:** 1 Research Institute for Stem Cell & Matters, Cell & Matter Corporation, Cheonan 31116, Republic of Korea; wisdom7970@gmail.com (J.-Y.Y.); drquangbinh@gmail.com (B.D.Q.); 2 Institute of Tissue Regeneration Engineering (ITREN), Dankook University, 119 Dandae-ro, Cheonan 31116, Republic of Korea; junheelee@dankook.ac.kr (J.H.L.); kimhw@dankook.ac.kr (H.-W.K.); 3 Department of Pediatric Dentistry, College of Dentistry, Dankook University, 119 Dandae-ro, Cheonan 31116, Republic of Korea; pedoshin@dankook.ac.kr (J.-S.S.); jbkim0222@dankook.ac.kr (J.-B.K.); 4 Department of Nanobiomedical Science & BK21 FOUR NBM Global Research Center for Regenerative Medicine, Dankook University, 119 Dandae-ro, Cheonan 31116, Republic of Korea; 5 UCL Eastman-Korea Dental Medicine Innovation Centre, Dankook University, 119 Dandae-ro, Cheonan 31116, Republic of Korea; 6 Mechanobiology Dental Medicine Research Center, Dankook University, 119 Dandae-ro, Cheonan 31116, Republic of Korea; 7 Cell & Matter Institute, Dankook University, 119 Dandae-ro, Cheonan 31116, Republic of Korea; 8 Department of Biomaterials Science, School of Dentistry, Dankook University, Cheonan 31116, Republic of Korea

**Keywords:** SHED, DPSC, hPL, cell therapy, stem cell characterization, standards for cell therapy, secretome, protein concentration, cost-effective method, cell proliferation, cell migration, self-renewal

## Abstract

SHEDs have demonstrated significant potential in cell therapy due to their superior proliferation rate, self-renewal and differentiation capacity (particularly neurogenesis attributed to their neural crest origin), and the less invasive procedure required for tissue collection compared to other stem cells. However, there is no established criterion to verify the minimum qualification to select one from numerous candidates, especially for SHEDs’ cultured FBS-free medium for clinic application. For that, we performed a characteristic analysis containing the growth rate, colony-forming unit (CFU) number, average colony size, and migration capacity with hPL-cultured SHEDs from 21 different donors, and we suggest the result as a minimum standard to filter out unqualified candidates. In addition, in the secretome analysis to predict the paracrine effect, it was found that upregulated proteins compared to the control were related to angiogenesis, immune response, and BMP signaling, and this was found to have a strong correlation only with protein concentration. This study presents a minimum standard for selecting cell therapy candidates and suggests the protein concentration of a conditioned medium as a cost-effective tool to expect the paracrine effect of SHEDs.

## 1. Introduction

For decades, research on stem cell therapy has steadily expanded, leading to numerous clinical trials for various diseases [1,2,3,4]. As a result, stem cells derived from various sources, including adipose tissue, bone marrow, and umbilical cords, have been actively investigated as candidate cells for stem cell-based therapy. SHEDs exhibit mesenchymal stem cell (MSC)-like characteristics, including a fibroblast-like morphology and adherence to plastic surfaces in culture, and the expression of key MSC surface markers (e.g., CD90, CD29, CD44, and CD73) [5,6]. In contrast, they lack hematopoietic markers such as CD34, or CD45. Beyond these characteristics, SHEDs possess several advantages, including minimal invasiveness, high proliferation rates, self-renewal capabilities, low immunogenicity, and remarkable differentiation potential, making them a promising cell source for stem cell therapy and regenerative medicine. In particular, their neural crest origin grants them strong neurogenic differentiation capabilities, highlighting their potential for various therapeutic applications [6,7,8,9,10,11,12,13,14].

When selecting the optimal cell source for clinical applications from numerous donors or evaluating the quality of cells as therapeutic agents, it is essential to assess several key parameters. First, it must be possible to obtain a sufficient number of cells with a low passage number, which is associated with high proliferation and self-renewal capacity. Second, the abilities of the cells to migrate to the target site and differentiate into the target cell type should be carefully considered, as they play a critical role in regeneration. Third, the secretome derived from stem cells, which influences angiogenesis, immune modulation, and differentiation, must also be evaluated [15,16,17,18,19,20,21]. Despite the importance of these criteria, there is currently no standard for assessing whether a cell source meets them.

Hence, we aimed to establish a standard for these basic parameters to assess the suitability of a cell source for cell therapy prior to evaluating its regenerative and therapeutic effects for specific targets.

To address these concerns, human platelet lysate (hPL) has emerged as a promising alternative and has been approved by the FDA for clinical applications. Platelets are essential cellular components in the bloodstream that play a key role in hemostasis, angiogenesis, and the regulation of innate immunity. Additionally, platelets store and release various growth factors and cytokines that regulate cell proliferation, differentiation, and tissue regeneration [22].

Building upon these regenerative properties, hPL contains an abundant supply of key growth factors, including PDGF, bFGF, TGF-β1, IGF-1, and VEGF, as well as a broad spectrum of cytokines and chemokines. Notably, these factors are present at higher concentrations compared to commonly used culture supplements such as FBS and human serum, thereby enhancing hPL’s ability to support cell proliferation and differentiation more effectively. Consequently, hPL holds strong clinical potential as a viable alternative to FBS for the culture of candidate cell sources intended for therapeutic applications.

By combining these properties, SHEDs cultured with hPL benefit from the bioactive factors derived from platelets, which enhance their proliferation and differentiation capacity. Utilizing these cells in cell therapy represents a strategic approach to maximizing the efficacy of stem cell-based treatments. Therefore, the integration of hPL and SHEDs is expected to play a crucial role in improving therapeutic efficiency through the combination of biological resources in the field of regenerative medicine [23,24,25].

Building on this understanding, in this study, we investigated the proliferation rate, self-renewal, and migration capacities of SHEDs derived from 21 different donors cultured in an hPL-containing medium. Additionally, secretome profiling through an antibody microarray was performed with the conditioned media of SHEDs and analyzed for correlation with the total protein amount and other parameters. This approach aims to establish a basic standard for qualifying SHEDs, particularly to infer paracrine effects efficiently and cost-effectively, given that a microarray analysis is relatively expensive and time-consuming.

## 2. Materials and Methods

### 2.1. The Primary Culture of SHED

Teeth were extracted and collected from 21 donors, aged between 6 and 12 years, following routine clinical extraction procedures at the Pediatric Department of Dankook University Dental Hospital.

SHEDs were isolated from the pulp according to a previously described method [24]. Briefly, the pulp was obtained by breaking the teeth with a hammer and then placing the pulp into microtubes. The pulp tissue was minced with fine scissors and subjected to enzyme digestion by incubation in 1 mL of 2 mg/mL collagenase (Collagenase type 1, LS004197, Worthington Biochemical Corp., Freehold, NJ, USA) solution and 1 mL of 4 mg/mL Dispase (Dispase II, D4693, Sigma-Aldrich, Merck, MO, USA) solution for 1 h at 37 °C in a water bath. The resulting pulp solution was filtered through a 70 µm strainer, centrifuged at 1500 rpm for 5 min, resuspended in the growth medium (GM: alpha MEM supplemented with 5% human platelet lysates (06961, STEMCELL Technologies, Vancouver, BC, Canada), 50 µg/mL Gentamicin (Gibco), 0.1 mM L-ascorbic acid phosphate (FUJIFILM Wako Pure Chemical Corporation, Osaka, Japan), and 1% Glutamax (Gibco, Thermo Fisher Scientific, Inc., Waltham, MA, USA)), and incubated at 37 °C in an atmosphere containing 5% CO_2_ and 95% humidity. The SHEDs were subcultured at 70–80% confluence. Stocks were prepared and stored in a liquid nitrogen tank at passage 2, and passage 4 cells were used for all experiments.

These procedures were conducted under approved ethical guidelines sanctioned by the Institutional Review Board of Dankook University Dental Hospital (approval number: DKUDH IRB-2021-12-002, date of approval: 23 December 2021). Written informed consent was obtained from the patients’ parents or legal guardians. Demographic details, including age, sex, and type of tooth (e.g., incisors, premolars), are summarized in Table 1.

### 2.2. Doubling Time and Growth Rate

In total, 50,000 SHEDs were seeded in a 60 mm dish. The cells were incubated and allowed to proliferate for 24, 48, and 72 h. At each time point, the number of cells was counted. For the determination of doubling time and growth rate, cell concentrations at 24 h and 72 h were used as the initial and final concentrations, respectively. The doubling time and growth rate were calculated using the following formulas [26]:

$$\mathrm{Doubling}\;\mathrm{time}=\frac{Duration\cdot \ln(2)}{\ln(\frac{Final\;concentration}{Initial\;concentration})}\;\;\mathrm{Growth}\;\mathrm{rate}=\frac{\ln(\frac{Final\;concentration}{Initial\;concentration})}{Duration}$$

### 2.3. Colony-Forming Units (CFU) Assay

For the CFU assay, 500 SHEDs were seeded in a 100 mm dish and cultured in GM. After 10 days, the cells were fixed with 4% PFA for 15 min, followed by staining with 2.5 mg/mL crystal violet (dissolved in 20% methanol) for 30 min. The number of colonies was counted after scanning the culture dishes. For the analysis of colony sizes, areas measuring 60 mm^2^ were randomly selected and colony sizes within these areas were measured using Fiji software (ImageJ 1.54f).

### 2.4. Cell Migration Assay

An 8 μm pore-sized 24-well insert was placed in a 24-well plate, and SHEDs were seeded in the upper chamber at a density of 4 × 10⁴ cells per 100 μL in alpha MEM. Next, 350 μL of growth medium (GM) was added to the lower compartment. After 24 h of incubation, cells attached to the insert were washed with Hank’s Balanced Salt Solution (HBSS) and then fixed with 4% paraformaldehyde (PFA). The cells were stained with 2.5 mg/mL crystal violet. Following washing with PBS, the non-migrated cells remaining on the upper surface of the insert were removed using a cotton swab. Hoechst staining was performed, and images were captured using a microscope. Cell numbers were counted using Fiji (ImageJ 1.54f).

### 2.5. Secretome Profiling

SHEDs were seeded with 1 × 10^6^ cells in a 100 mm dish in the growth medium. After 24 h, the medium was removed completely and washed with plain alpha MEM three times. Next, 5 mL of plain alpha MEM (with 1% P/S) was added and incubated for the next 24 h. After that, the conditioned medium was collected and filtered with a 0.2 μm syringe filter. Another dish contained a serum-free medium and was used for the negative control. The antibody microarray was performed by Ebiogen. In brief, the concentration and purity of the purified sample were confirmed using a BCA protein assay kit (Pierce, Rockford, IL, USA) and UV absorbance measurement with the MultiSkan FC (Thermo Fisher Scientific, Waltham, MA, USA). For the antibody microarray, the RayBiotech slide was dried, blocked, and incubated with the sample, followed by sequential washes with 1X wash buffer I and II. It was then treated with biotin-conjugated anti-cytokine antibodies and Cy3-conjugated streptavidin, washed again, and rinsed with de-ionized water. Data acquisition was completed using a GenePix 4100A Scanner, and an analysis was performed with GenePix Software (Genepix Pro 6.0), UniProt database (Available online: https://www.uniprot.org/ (accessed on 20 December 2023)), and ExDEGA (v.5.2.1, Ebiogen Inc., Seoul, Republic of Korea) for data mining and visualization. The secretome factor for each sample was calculated as the sum of the fold changes from proteins upregulated by more than 2.5-fold compared to the control (blank media), based on the proteomic analysis.

### 2.6. Statistical Analysis

All statistical analyses were performed using GraphPad Prism software (version 9.4.1). Data normality was assessed using the Shapiro–Wilk test. A one-way ANOVA followed by Dunnett’s multiple comparisons test were used to compare mean values and identify significant differences between individual groups and the overall mean. Pearson’s correlation coefficient (*r*) with two-tailed *p*-values was calculated to evaluate the relationships between variables, and scatter plots with fitted lines were generated for visual representation. Data are presented as mean ± standard deviation (s.d.), with significance levels indicated as *p* < 0.05, *p* < 0.01, *p* < 0.001, and *p* < 0.0001. Correlation matrices were constructed to represent both positive and negative relationships among variables. Statistical significance for correlations with *p* < 0.0001 was indicated with an asterisk (*) in the heatmap.

## 3. Results

### 3.1. Proliferation Capacity Analysis

**Figure 1:**
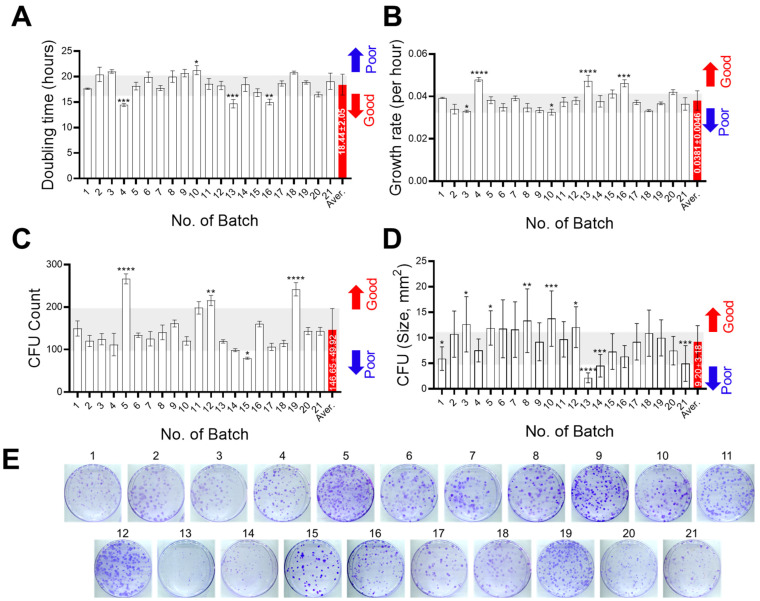

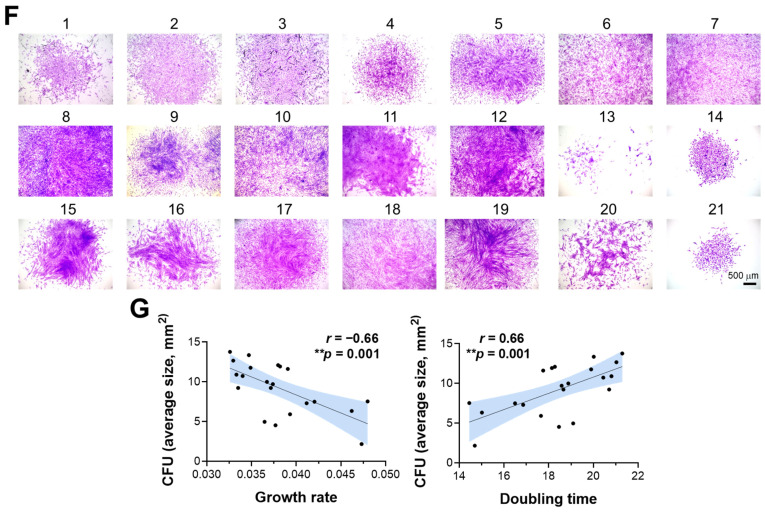
Proliferation and self-renewal capacity analysis of SHEDs derived from 21 different donors. (**A,B**) Doubling times (**A**) and growth rates (**B**) were calculated based on cell counts at 24 and 72 h post 50,000 cell seeding. (**C**,**D**) Results from colony formation unit (CFU) assay show colony numbers (**C**) and average colony sizes (**D**) measured 10 days after seeding 500 cells. The light gray area and red bar in panels (**A**–**D**) indicate the average ± standard deviation (s.d.), with values above or below the average categorized as Good (red) or Poor (blue). (**E**,**F**) Representative images of CFU assays are shown at low magnification ((**E**), full 100 mm dish scan) and high magnification (**F**). Colonies were stained with crystal violet for visualization. Scale bar: 500 μm. (**G**) Correlation plots with linear regression showing the relationship between colony sizes and growth rates (left) or doubling times (right). The shaded blue area represents the 95% confidence interval (CI) for the regression line. Pearson’s correlation coefficient (r) values and two-tailed *p*-value are indicated (*p*). (**A**–**D**) Data are presented as mean ± s.d. (n = 3), with statistical significance determined using one-way ANOVA followed by Dunnett’s multiple comparisons test. Significance levels: * *p* < 0.05, ** *p* < 0.01, *** *p* < 0.001, **** *p* < 0.0001.

SHEDs from 21 different donors were analyzed for proliferation capacity by measuring doubling time and growth rate. Cells were counted at 24 (initial point) and 72 (final point) hours after seeding to determine doubling time and growth rate (Figure 1A,B). The average of the doubling times was 18.44 ± 2.05 h and that of growth rate was 0.0381 ± 0.0046 per hour. Among the samples, most SHEDs fell within the average range, and three SHEDs exhibited significantly lower doubling times (with one notably around 14 h), while one showed a higher value but still within 24 h. The growth rate showed similar patterns with doubling time. Additionally, the growth rate between 24 h and 48 h was calculated and compared to analyze the changes in proliferation rate over time (Figure S1). The analysis revealed that cell numbers consistently increased over time across all batches (Figure S1A,B). However, while the growth rate remained largely stable in most batches, a slight decline was observed in certain batches, including batch 4 and batch 13 (Figure S1C).

### 3.2. Self-Renewal Capacity Analysis

To evaluate self-renewal capacity, a CFU assay was performed. The average colony number was 146.65 ± 49.92 and most samples fell within this range, with three samples showing significantly higher numbers and one sample lower than average. The size of the colonies varied greatly compared to the colony numbers even in same samples, and colony density was also varied, with some colonies being sparse and others dense, despite having similar colony numbers (Figure 1C–F). A correlation analysis was conducted between colony size and growth rate or doubling time. The results showed that the average colony size decreased with an increasing growth rate (r = −0.66, *p* = 0.001) and a decreasing doubling time (r = 0.66, *p* = 0.001) (Figure 1G). However, colony number was not correlated with growth rate and doubling time (r value are below 0.1 or over −0.1) (Figure S2).

### 3.3. Migration Capacity Analysis

**Figure 2:**
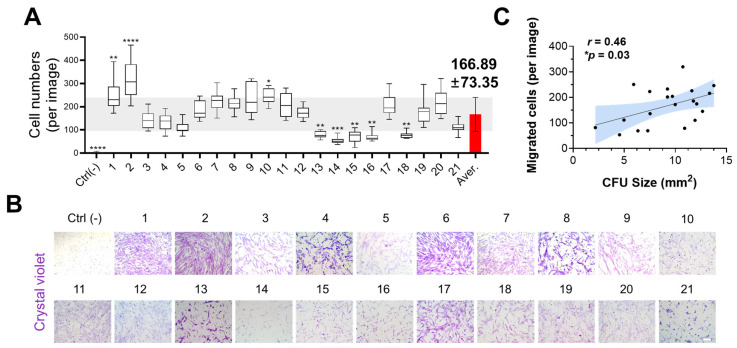
Migration capacity analysis of SHEDs derived from 21 different donors. (**A**,**B**) SHED (40,000 cells per well) were seeded in the upper chamber of a transwell insert and allowed to migrate for 24 h. (**A**) Quantification of migrated cell numbers based on DAPI-stained images. The red bar and the light gray area indicate the average ± standard deviation (s.d.). Statistical significance was determined using one-way ANOVA followed by Dunnett’s multiple comparisons test against the average. Data are presented as mean ± s.d. (* *p* < 0.05, ** *p* < 0.01, *** *p* < 0.001, **** *p* < 0.0001). (**B**) Representative images of migrated cells stained with crystal violet (upper). Scale bar: 100 μm. (**C**) Correlation plots with linear regression showing the relationship between colony size and migrated cell numbers. The shaded blue area represents the 95% confidence interval (CI). Pearson’s correlation coefficient (r = 0.46) and *p*-value (*p* = 0.03) were used to evaluate statistical significance.

To evaluate migration ability, we used a transwell membrane array. Migrated cells were quantified using DAPI-stained images. On average, 166.89 ± 73.35 cells migrated per image (100× magnification) (Figure 2A,B and Figure S3). Notably, the number of migrated cells was positively correlated with colony size (r = 0.46), but there was no correlation with colony numbers (Figure 2C and Figure S4).

### 3.4. Secretome Analysis

We analyzed the secretome in the conditioned medium (CM) using an antibody microarray (Figure S6). Before analysis, protein concentrations were measured by the Bradford assay, revealing that some samples had undetectable levels (#2 and #13), while most exhibited diverse individual expressions. Notably, samples #17 and #18 exhibited significantly high protein levels, nearly four times the average (Figure S5A). The protein amount did not correlate with either colony number or colony size (Figure S5B,C).

**Figure 3:**
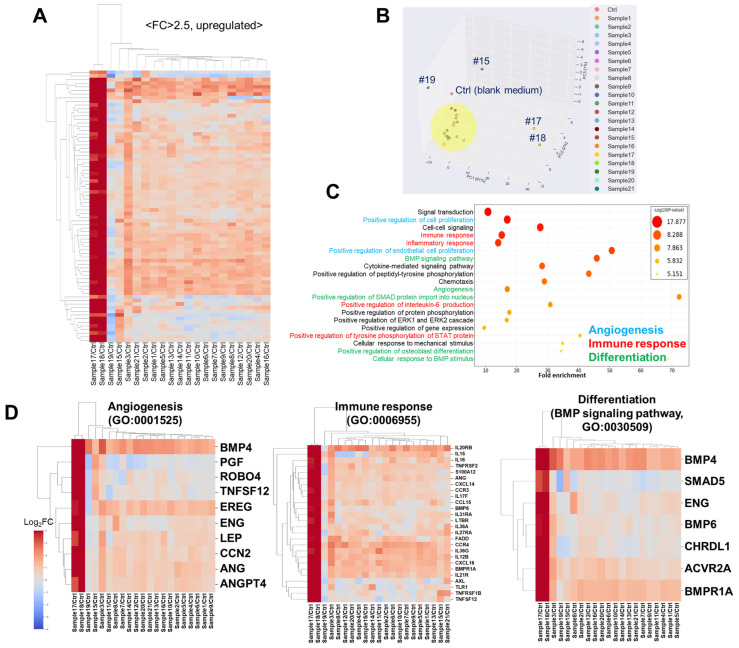
Secretome analysis using conditioned media from SHEDs derived from 21 different donors. Differentially secreted proteins relative to blank (FC > 2.5, normalized value (log2) > 2) were analyzed. (**A**) Heatmap of secretome. (**B**) PCA analysis: #15, #17, #18, and #19 show distinct characteristics compared to others (yellow circle). (**C**) Biological process gene ontology from David analysis, with particularly interesting biological processes marked with different colored lines. (**D**) Heatmap of secretome from 21 different donors (FC > 2.5, normalized value (log2) > 2) assigned to angiogenesis, immune response, and BMP signaling pathway (as representative differentiation). FC: Fold change.

Differentially secreted factors in the samples, compared with the control (plain alpha MEM without cells), were displayed in a heatmap with an adjusted fold change >2.5 and normalized value (log2) > 2 (Figure 3A). The PCA analysis indicated that most groups were similar, except for samples #15, #17, #18, and #19 (Figure 3B). A gene ontology (GO) enrichment analysis highlighted the potential roles of secretome in angiogenesis, immune response, and differentiation. As shown in Figure 3D, differentially enriched secreted factors were related to angiogenesis, immune response, and differentiation, particularly BMP signaling.

**Figure 4:**
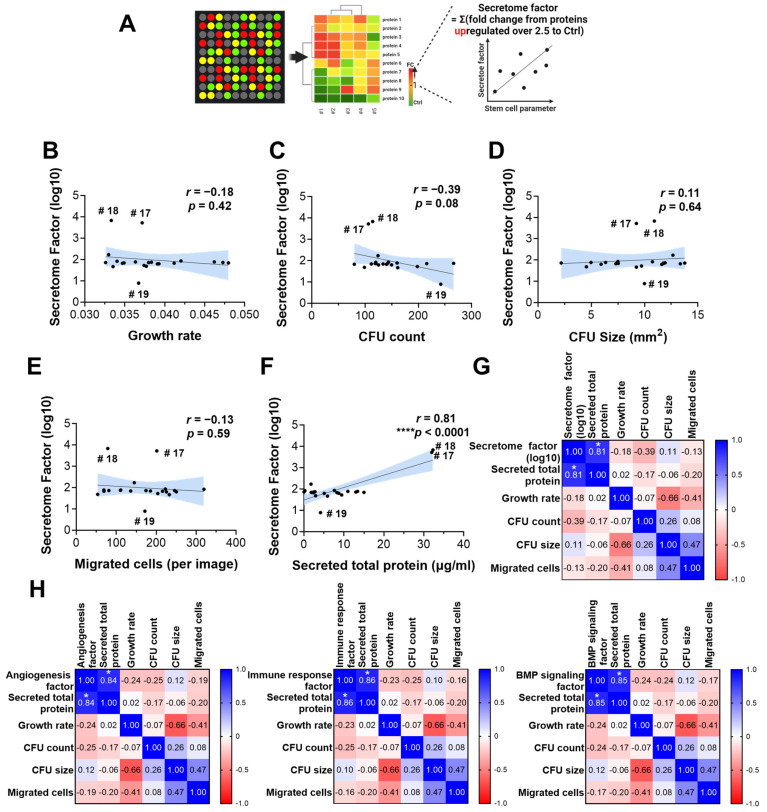
Total protein amounts from conditioned media can predict general secretome factors. (**A**) Schematic summary of secretome factor calculation. Fold changes of proteins upregulated over 2.5-fold compared to blank medium were summed to calculate the secretome factor. (**B**–**F**) Correlation plots with linear regression showing the relationship between secretome factor and various stem cell parameters including growth rate, CFU number, average colony size, migrated cell numbers, and total secreted proteins amounts. The blue shaded areas represent the 95% confidence interval of the regression line, illustrating the uncertainty in the correlation. Pearson’s correlation coefficient (r) was used to evaluate relationships, and statistical significance was assessed with two-tailed *p*-values. (**G**) Pearson’s correlation matrix showing relationships among the secretome factor, total protein amounts, and stem cell parameters. Only the total protein amount showed significant correlation with the secretome factor (*, *p* < 0.0001). (**H**) Pearson’s correlation matrix depicting associations between secretome factors and stem cell parameters. Total protein amount showed significant positive correlation with angiogenesis factors, immune response factors, and BMP signaling factor (*, *p* < 0.0001).

To identify a simple and cost-effective method for estimating the secretome without using microarrays, we analyzed the correlation between the secretome factors and other parameters, including cell proliferation, colony formation, migration, and protein concentration measurements. Three samples (#3, #17, #18) showed significantly higher secretome levels compared to the control, but this did not correlate with better capacity in other factors. Only protein concentration was strongly correlated with secretome factor levels (r = 0.86) and detailed factor levels related to angiogenesis, immune response, and BMP signaling (Figure 4A–H). This indicates that secretome levels, particularly those involving angiogenesis, immune response, and BMP signaling, can be estimated by measuring protein concentration.

## 4. Discussion

Despite the growing demand for SHEDs, there are no reported criteria to ensure that candidate cells obtained from multiple donors meet the minimum qualifications for cell therapy.

To address this need, we collected SHEDs from 21 donors and conducted a series of evaluations with them cultured in media containing hPL, a substitute for the animal-derived protein FBS, to reduce immune response in clinical applications [24,27,28]: (1) a proliferation test to determine whether a sufficient number of cells could be obtained with minimal passage, (2) a CFU assay to assess self-renewal ability, (3) a migration test to assess the ability to migrate to the target tissue, and (4) an analysis of cytokine secretion related to angiogenesis, immunomodulation, and differentiation, which are crucial factors for regenerative or therapeutic outcomes. Through these tests, we aimed to establish standards for evaluating SHED suitability for therapeutic applications.

The doubling time, which measures how long it takes for a cell population to double in number, was found to be 18.44 ± 2.05 h (measured from 24 to 72 h). Concurrently, the growth rate, which indicates how quickly the cell population increases over time, was observed to be 0.0381 ± 0.0046 per hour during this period. Based on the analysis of these results, only one sample (#10) failed to meet the qualification criteria, while three samples (#4, #13, and #16) showed significantly higher capacities compared to the average. We examined various parameters, including the tooth type, donor age, and sex, to identify potential reasons for these observations. However, no specific differences were noted when compared to other samples. For instance, sample #10, which showed the lowest proliferation capacity, originated from the SNT of a 7-year-old male, the same as sample #4, which exhibited one of the highest proliferation capacities. This suggests that donor age, sex, and tooth location may not be determining factors of cell proliferation capacity.

We initially anticipated that larger pulp tissues would yield a greater number of isolated cells, potentially influencing evaluation parameters such as proliferation. However, this trend was not observed. Interestingly, smaller pulp tissues sometimes yielded a higher number of cells. This inconsistency is likely due to intrinsic functional characteristics of pulp tissue, such as cell density.

In this study, we did not directly analyze the correlation between pulp weight and outcomes. Pulp tissue is inherently very light and exists in small quantities, making it difficult to detect differences without precise measuring instruments. Furthermore, we determined that such minor differences in weight were unlikely to cause functional differences in the cells, which is why weight was excluded as a parameter in this evaluation. Nonetheless, this observation highlights the possibility that intrinsic factors, such as cell density or biological composition, may play a more critical role in determining cell yield and functionality compared to physical parameters like size or weight. Such a lack of correlation was similarly observed in self-renewal, migration, and secretome assays.

In addition, we analyzed colony-forming units (CFUs) to evaluate self-renewal capacity. A high CFU count indicates that stem cells can maintain their undifferentiated state and produce a greater number of progenitor cells, essential for tissue formation and healing [29,30,31]. Therefore, the CFU count serves as a criterion for evaluating stem cell quality. Colony number reflects the ratio of cells to maintain their self-renewal capacity, while colony size indicates their proliferation rate. We performed CFU assays and measured both colony number and size. Most samples formed colonies of average size and number, whereas a few samples produced smaller (#13, 14, and 21) and fewer (#15) colonies. As with the proliferation test, we could not find any differences in parameters among samples. Furthermore, we analyzed the correlation between the CFU results and growth rate or doubling time to assess the relationship between proliferation and self-renewal capacity. As expected, colony size showed a strong correlation with the growth rate (*r* = −0.66, *p* = 0.001) and doubling time (*r* = 0.66, *p* = 0.001), indicating a link to the proliferation rate. However, colony numbers did not correlate with these parameters.

The ability of stem cells to home to target tissues (damaged or diseased areas) by passing through the extracellular matrix or tissue environment is crucial for enhancing regenerative potential [32,33,34]. Hence, we performed a migration test toward the growth medium (acting as a chemotactic signal) using a transwell membrane. However, cell migration was not uniform across the entire area, which may be attributed to heterogeneity in cell attachment, even though we made efforts to ensure even cell seeding. To provide a more comprehensive representation of the migration pattern, we included a broad range of areas (n = 15) in our analysis. As a result, three samples (#1, #2, and #10) showed significantly higher migrated cell numbers and five samples (#13, #14, #15, #16, and #18) could not satisfy the criterion. Interestingly, samples #13 and #14 were not quantified in a self-renewal capacity (especially colony size) or migration test. This result expanded the notion that the number of migrated cells was slightly correlated (*r* = 0.46, *p* = 0.03) with colony size (not with colony number).

Next, a secretome analysis was conducted using a conditioned medium (CM) collected over 24 h with a serum-free medium (excluding hPL). Secretome factors such as cytokines and growth factors from stem cells play pivotal roles in various biological processes, including cell signaling, immune modulation, cell proliferation, angiogenesis, and differentiation [35,36,37,38,39,40]. A gene ontology analysis of differentially secreted factors (upregulated) in CM revealed that these factors are mostly related to these biological processes.

It is well known that one of the critical determinants of success in tissue regeneration and repair through stem cell transplantation is the paracrine effect of stem cell-secreted cytokines and growth factors. While their concentrations can be measured through antibody microarrays (broad protein) or ELISA (specific), these methods are both costly and time-consuming.

Therefore, we analyzed correlations between the microarray results and protein amount, proliferation, and migration. We found that only the protein amount strongly correlated with the secretome results, which included factors related to angiogenesis, immune response, and BMP signaling. This indicates that the individual contributions within the secretome are relatively consistent, leading to strong correlations between them. In conclusion, we can predict the secretome’s effects, particularly concerning angiogenesis, immune response, and BMP signaling, by measuring protein concentration. These findings provide a foundation for future functional studies to validate the specific roles of individual secreted factors. While this study primarily characterizes the secretome composition of SHEDs cultured in hPL, further investigations using gain-of-function and loss-of-function experiments will be valuable in elucidating the mechanistic contributions of key secreted factors to angiogenesis, immune modulation, and differentiation. Such studies could enhance our understanding of the paracrine effects of SHEDs and their therapeutic potential in regenerative medicine.

Although we used cells derived from as many donors as possible to establish this standard, there are still limitations. Variations in experimenter technique, hPL quality depending on the manufacturer, donor ethnicity, and other factors may influence the applicability of experimental results to this standard. Nevertheless, we established this standard because hPL is widely recognized as one of the most reliable human-derived FBS substitutes for cell therapeutics intended for clinical research and trials, and SHEDs are not only readily obtained from young donors but have also demonstrated superior capabilities compared to other stem cells. To address these limitations, further studies are required to assess whether this standard can be applied across various components, including other human-derived alternatives and widely used commercial media.

## 5. Conclusions

Despite the growing demand for SHEDs for cell therapy, there are no reported criteria to ensure that SHED candidates obtained from multiple donors meet the minimum qualifications for cell therapy. For that, here we provide the summarized characteristic results including proliferation, migration, self-renewal, and secretome capacity from 21 donor-derived SHEDs cultured in xeno-free media (hPL-contained media) (Table 2) and suggest a standard to judge the adequacy of the candidate.

**Figure 5:**
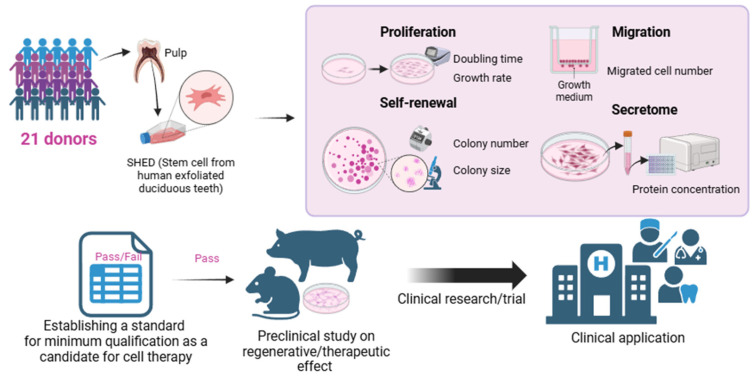
Schematic image of the current study. We conducted assays of proliferation, self-renewal, and migration on SHEDs obtained from 21 different donors. Additionally, we found that the secretome concentration related to angiogenesis, immunomodulation, and differentiation could be predicted by protein concentration. Based on these results, we propose criteria for the minimum qualification as a candidate for cell therapy. Cell candidates passing these criteria can undergo further studies, including preclinical studies, clinical research, and clinical trials, for eventual clinical application.

In addition to this, we can predict the secretome’s effects on angiogenesis, immune response, and BMP signaling by measuring protein concentration. These findings establish a foundation for the cost-effective evaluation of its suitability as a SHED candidate for therapeutic applications. In conclusion, by screening candidates based on their compliance with the established minimum qualification standards, we can streamline the selection process for subsequent efficacy evaluations, clinical research, and trials, ultimately advancing toward the goal of clinical application (Figure 5). This approach will not only minimize costs but also address challenges associated with the preparation of source cells, while maximizing regenerative and therapeutic efficacy.

## Figures and Tables

**Table 1:** Donor Demographics and Extracted Teeth.

Donor ID	Sex	Age (Years)	Tooth Type	Donor ID	Sex	Age (Years)	Tooth Type
**1**	M	8	SNT	**12**	M	11	SNT
**2**	M	7	SNT	**13**	F	7	SNT
**3**	M	10	Canine	**14**	F	7	SNT
**4**	M	7	SNT	**15**	M	6	SNT
**5**	F	6	Incisor	**16**	M	7	SNT
**6**	F	11	SNT	**17**	M	11	Premolar
**7**	M	7	SNT	**18**	M	6	SNT
**8**	M	8	SNT	**19**	M	6	SNT
**9**	F	7	Incisor	**20**	F	12	Canine
**10**	M	7	SNT	**21**	M	8	SNT
**11**	F	6	Canine				

M: Male, F: Female, SNT: Supernumerary Tooth.

**Table 2:** Standard table for stem cell evaluation items.

Evaluation Item (Unit, Period)	SHEDs Standard (n = 21)
Doubling time (h, 24 to 72 h)	18.44 ± 2.05
Growth rate (per h, 24 to 72 h)	0.0381 ± 0.0046
CFU count (in 57.50 cm^2^,10 days)	146.65 ± 49.92
Colony size (mm^2^, 10 days)	9.20 ± 3.18
Migration ability (per image-100×, 24 h)	166.89 ± 73.35

## Data Availability

The data used in this article are available from the corresponding author upon reasonable request.

## Supplementary Materials

The following supporting information can be downloaded at: https://www.mdpi.com/article/10.3390/cells14040316/s1, Figure S1: Progressive cell proliferation with a steady growth rate; Figure S2: Scatter plots with Pearson’s correlation coefficient (r) values and two-tailed *p*-value (*p*) between colony numbers and growth rate or doubling time; Figure S3: Scatter plots with Pearson’s correlation coefficient (r) values and two-tailed *p*-value (*p*) between colony numbers and migrated cell numbers; Figure S4: Nuclear staining of migrated SHED using DAPI; Figure S5: (A) Protein amounts measured by Bradford assay and (B, C) Scatter plots with Pearson’s correlation coefficient (r) values and two-tailed *p*-value (*p*) between protein concentration (μg/mL) colony numbers or colony sized (mm^2^); Figure S6: Scan images analyzed by Antibody array.

## Abbreviations

ANOVA	Analysis of variance
bFGF	Basic fibroblast growth factor
BMP	Bone morphogenetic protein
CI	Confidence interval
CM	Conditioned medium
CFU	Colony-forming unit
DAPI	4′,6-Diamidino-2-phenylindole
ECM	Extracellular matrix
GM	Growth medium
GO	Gene ontology
hPL	Human platelet lysate
IHC	Immunohistochemistry
IGF-1	Insulin-like growth factor-1
MEM	Minimum essential medium
MSCs	Mesenchymal stem cells
PCA	Principal component analysis
PDGF	Platelet-derived growth factor
SHEDs	Stem cells from human exfoliated deciduous teeth
SNT	Supernumerary tooth
TGF-β1	Transforming growth factor-beta 1
VEGF	Vascular endothelial growth factor
