# Supplementary material for: Establishing Minimum Criteria for Stem Cells from Human Exfoliated Deciduous Teeth (SHEDs) Cultured in Human Platelet Lysate (hPL)-Contained Media as Cell Therapy Candidates: Characterization and Predictive Analysis of Secretome Effects
Source: Cells. 2025 Feb 19;14(4):316. doi: 10.3390/cells14040316 (PMC11854447; doi:10.3390/cells14040316)
Supplement: Supplementary file 1 [file cells-14-00316-s001.zip › cells-3411479-supplementary.pdf]

Supplementary data

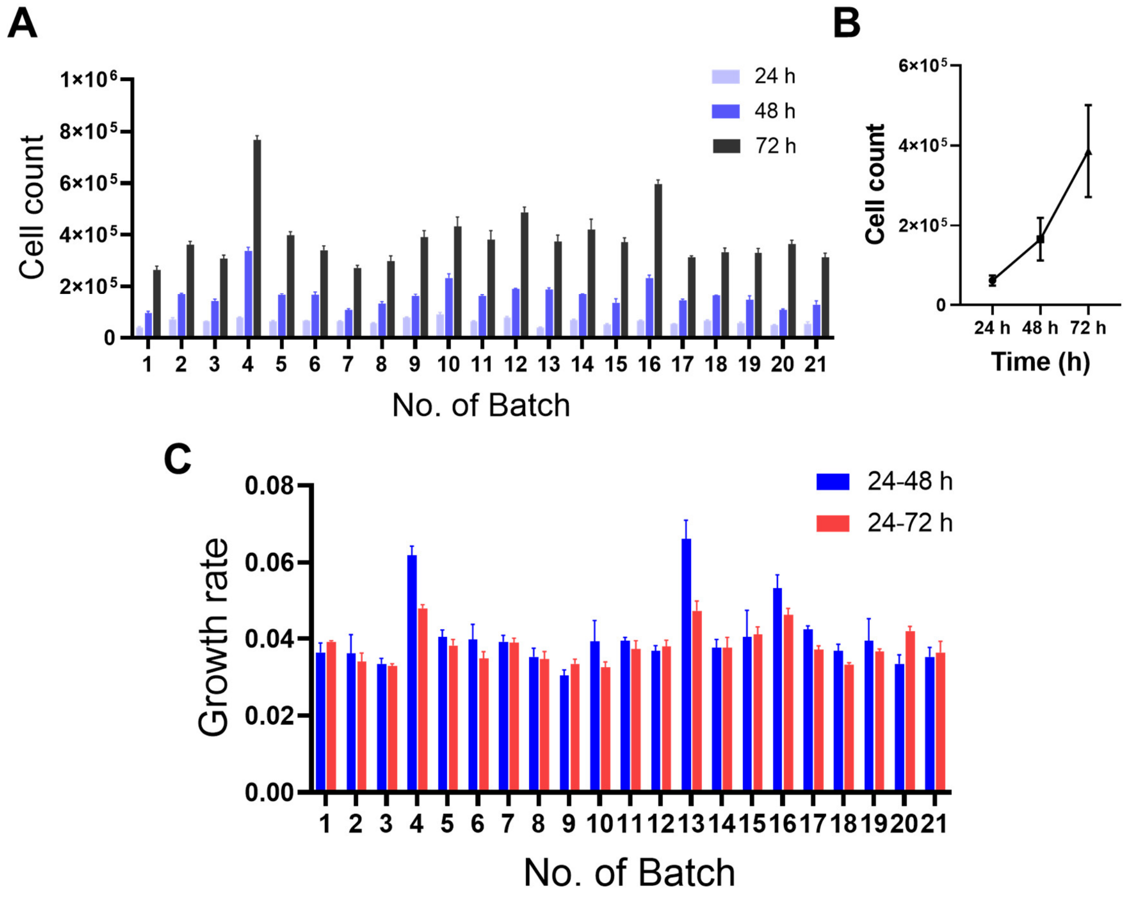

Figure S1. Progressive cell proliferation with a steady growth rate. A) Cell count at 24, 48, and 72 h for each batch. B) Mean ± S.D. of cell counts at different time points. C) Growth rate comparisons between 24-48 h and 24- 72 h.

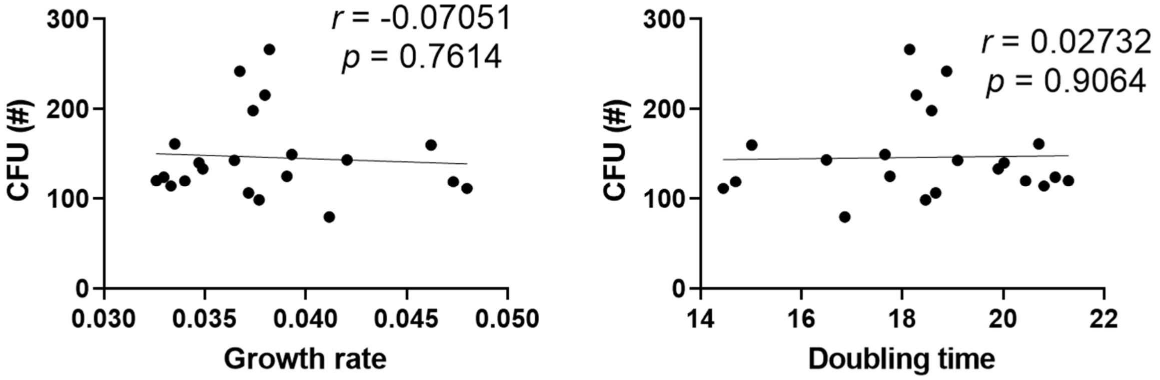

Figure S2. Scatter plots with Pearson`s correlation coefficient (r) values and two-tailed *p*-value (*p*) between colony numbers and growth rate or doubling time.

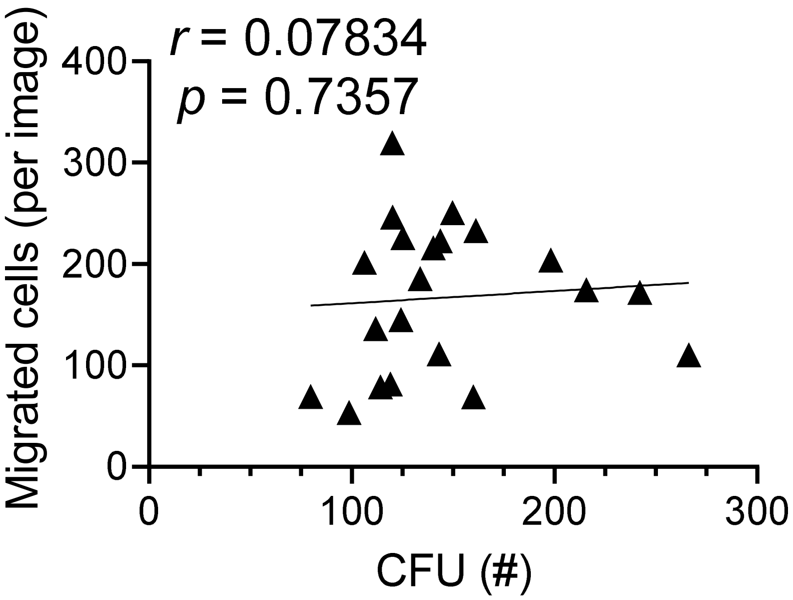

Figure S3. Scatter plots with Pearson`s correlation coefficient (r) values and two-tailed *p*-value (*p*) between colony numbers and migrated cell numbers.

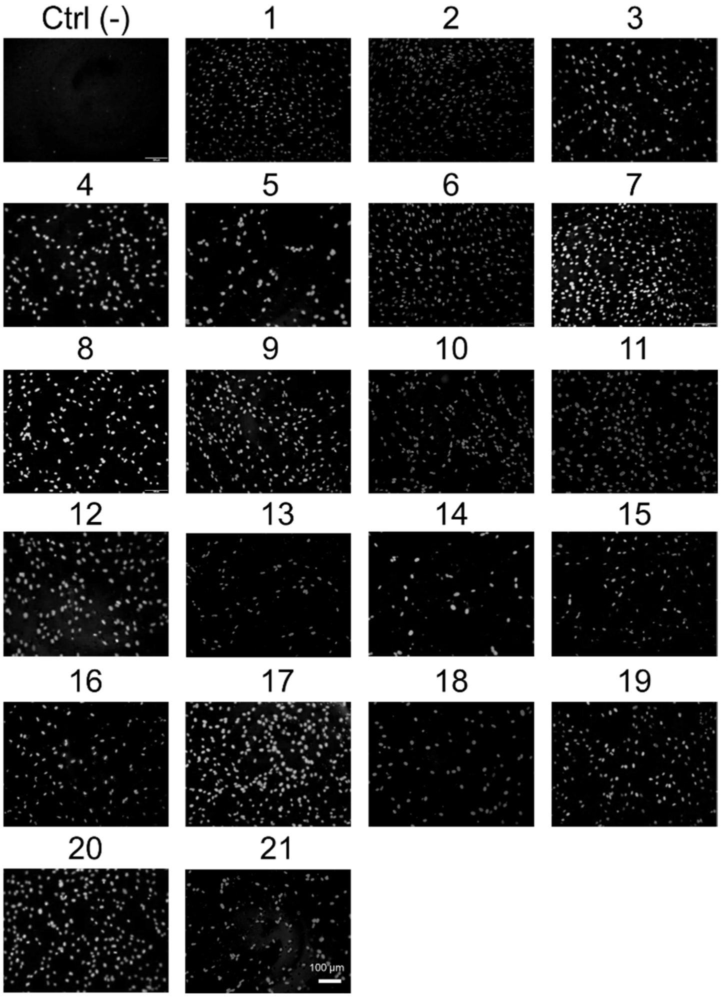

Figure S4. Nuclear staining of migrated SHED using DAPI. Representative images of migrated SHED derived from 21 different donors stained with DAPI for migrated cell counting. Cells were seeded in the upper chamber of a transwell insert (40,000 cells per well) and allowed to migrate for 24 hours. After migration, cells were fixed and stained with DAPI, and images were acquired for quantification of migrated cells (n=15). Scale bar: 100 µm.

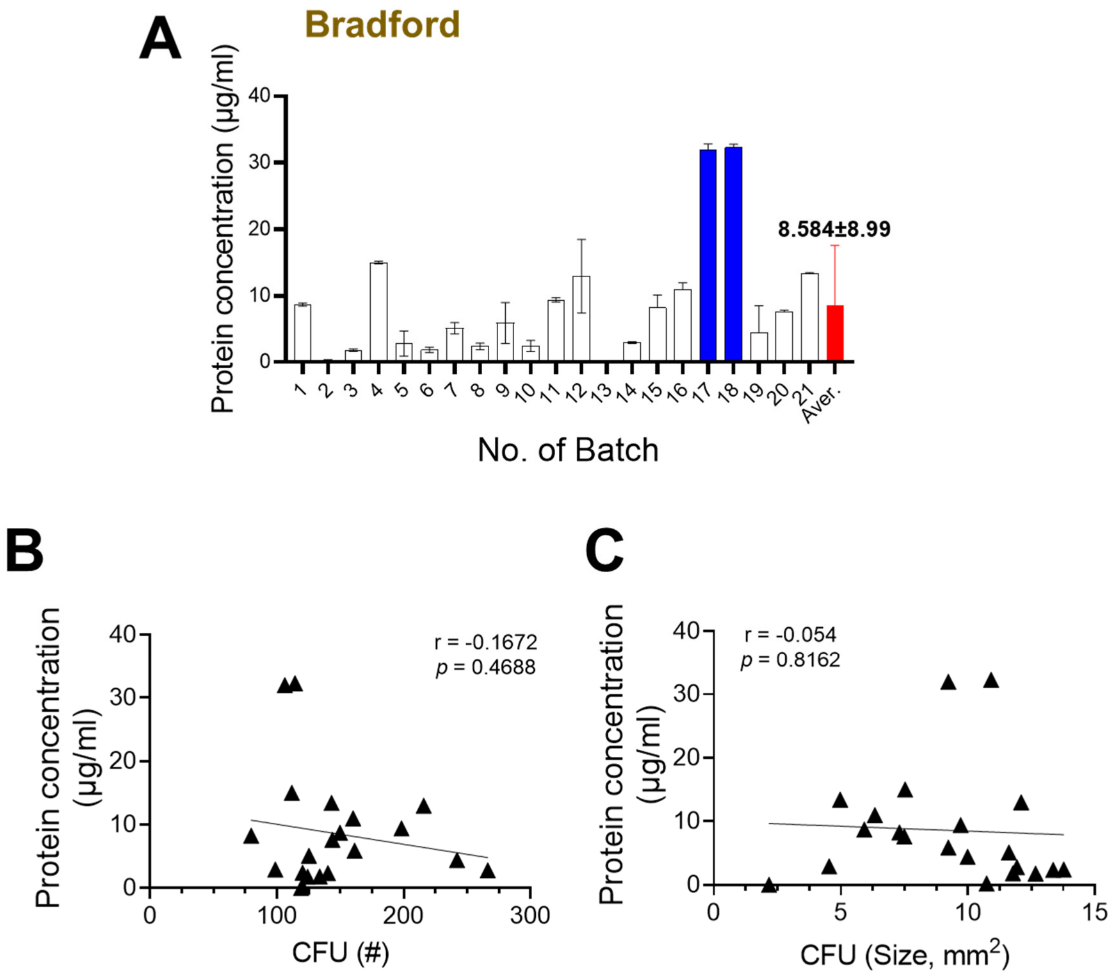

Figure S5. (A) Protein amounts measured by Bradford assay and (B, C) Scatter plots with Pearson`s correlation coefficient (r) values and two-tailed *p*-value (*p*) between protein concentration (μg/ml) colony numbers or colony sized (mm^2)

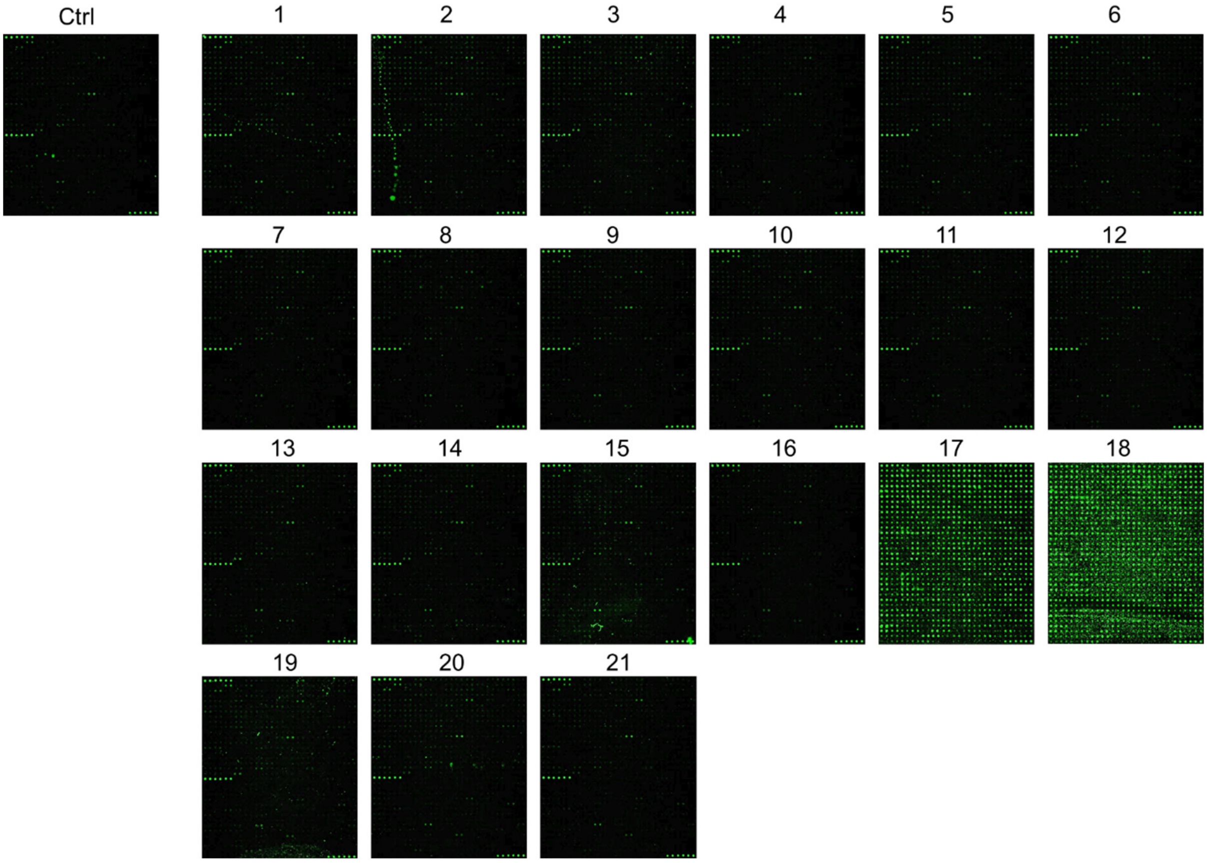

Figure S6. Scan images analyzed by Antibody array
